# Navigating the Role of Surgery in Optimizing Patient Outcomes in Traumatic Brain Injuries (TBIs): A Comprehensive Review

**DOI:** 10.7759/cureus.71234

**Published:** 2024-10-10

**Authors:** Meenakshi Reddy Yathindra, Nagma Sabu, Seetha Lakshmy, Celine A Gibson, Alexander T Morris, Sumaiya Farah Fatima, Aarushi Gupta, Lilit Ghazaryan, Jean C Daher, Grace Tello Seminario, Tanvi Mahajan, Humza F Siddiqui

**Affiliations:** 1 Internal Medicine, Kasturba Medical College, Mangalore, Mangalore, IND; 2 Surgery, Jonelta Foundation School of Medicine University of Perpetual Help System DALTA, Las Pinas City, PHL; 3 Internal Medicine, Amala Institute of Medical Sciences, Thrissur, IND; 4 Kinseology, Acadia University, Wolfville, CAN; 5 Medicine, Burrell College of Osteopathic Medicine, Las Cruces, USA; 6 Medicine and Surgery, Dr VRK Women's Medical College, Aziznagar, IND; 7 Medicine, Avalon University School of Medicine, Youngstown, USA; 8 Medicine, Yerevan State Medical University, Yerevan, ARM; 9 Medicine, Lakeland Regional Health, Lakeland, USA; 10 Medicine, Universidad de Ciencias Medicas, San Jose, CRI; 11 Medicine and Surgery, Cayetano Heredia Peruvian University, Lima, PER; 12 Internal Medicine, Maharishi Markandeshwar Medical College and Hospital, Solan, IND; 13 Internal Medicine, Jinnah Postgraduate Medical Centre, Karachi, PAK

**Keywords:** contusions, cranioplasty, craniotomy, decompressive craniectomy (dc), epidural hematoma, external ventricular drain (evd), glasgow coma scale (gcs), intracranial pressure (icp), subdural hematoma, traumatic brain injury (tbi)

## Abstract

Traumatic brain injuries (TBIs) present with symptoms ranging from a mildly altered level of consciousness to irreversible coma and death. The most severe stage of TBIs is diffuse axonal injury and swelling affecting the whole brain. Management strategies are based on the classification of TBIs by severity and type and range from cognitive therapy sessions to complex surgeries. Neuroimaging modalities, predominantly magnetic resonance imaging, and the clinical Glasgow Coma Scale are principal indicators to diagnose and assess a patient’s condition and neurological status and decide optimal treatment modality. In this review, we have summarized the indications and patient outcomes based on neurological and functional status, post-surgical complications, and mortality rates for various life-saving interventional procedures including surgery for brain contusions, intracranial hematomas and penetrating injuries, and craniectomy and ventriculostomy for elevated intracranial pressure and hydrocephalus. Cranioplasty performed for aesthetic purposes has also been explored. Overall quality evidence presented advocates surgery as needed for improved patient outcomes resulting in early recovery and decreased mortality, especially with the emergence of minimally invasive techniques. However, there is still an increased risk of certain complications like infections and bleeding and severe disabilities leading to a vegetative state with surgery. Some guidelines have been formed to provide indications for optimal management of TBI patients including surgeries, although their effectiveness in each individual case is debatable. It is imperative to explore certain key areas like the timing of the surgery and the role of intensive patient monitoring pre- and post-procedure in future studies and lay down guidelines also applicable to resource-limited areas. Also, a deeper understanding of physiological and pathological mechanisms of functional outcomes post-surgery will help clinicians predict the patient’s course of recovery.

## Introduction and background

A traumatic brain injury (TBI) is a common morbidity known for its poor outcomes in severe cases [1]. It is a leading cause of mortality and disability worldwide. In 2018, the global incidence was estimated to be 69 million cases, including 5.48 million cases deemed severe [2,3]. Out of all traumas, TBIs cause the most mortality and disability, and nearly one-third of severe TBI patients result in mortality [3,4]. Incidences remain highest in North America with 1299 cases per 100,000 compared to Africa with the lowest at 801 cases per 100,000 [2]. Due to the severity and heterogeneous nature of moderate to severe TBIs, outcomes vary widely in terms of types of disability. Overall moderate to severe TBI outcomes include death, varying degrees of disability, vegetative state, or complete recovery [5].

The treatment and management are largely based on the classification of TBIs by severity and type. Classification modalities use both clinical and neuroimaging scales. Severity is most commonly interpreted by the clinical Glasgow Coma Scale (GCS) as it is both simple and easily repeatable. It relies on rating ocular, verbal, and motor responses with a total magnitude ranging from 3 to 15. Traditionally, a GCS of 13-15 is reported as mild, 9-12 as moderate, and <8 as severe [6]. A drawback of the GCS is confounding clinical factors, secondary to either medically induced sedation or another cause of non-neurological altered mental status, that are not quantitatively differentiated. To mediate this, an alternative clinical scoring system, the Full Outline of Un-Responsiveness (FOUR), was developed. However, FOUR’s GCS equivoques and reproducibility by non-neurologists still remain in question [6,7]. Injury types can be classified by scales utilizing the CT-based Marshall neuroimaging and include the Marshall scale which classifies injuries into diffuse (four categories) and evacuated (two categories). It is used by neurotrauma centers to predict the risk of increased intracranial pressure (ICP). Another CT scale, the Rotterdam scale, was developed to overcome the limitations of the Marshall scale as a predictive measure and can independently predict outcomes for TBI patients. It is widely used in neurotrauma centers for its improved sensitivity and specificity in predicting outcomes [8,9].

TBIs are distinct from other neurological pathologies in that an external force precipitates an acute disruption of normal brain function [10]. The pathophysiology of a TBI is classically divided into two periods, one for primary brain injury and one for secondary brain injury [11]. Primary brain injury is the result of the precipitating force in one of the following categories: shearing mechanisms, contusions (most common), and extra-axial (hemorrhage and hematoma) [5]. The result of a primary brain injury is that axon membranes are directly damaged by causative force [12]. Secondary brain injury is the result of a cascade of molecular injury mechanisms that includes many mediators including ischemia, hypoxia, electrolyte imbalances, neurotransmitter release, and local inflammatory responses [5,12].

Critical to the optimal outcome of moderate to severe TBIs are early intervention and continuity in care from prehospital, hospital, neurosurgery, and intensive care management teams collectively with the mainstay goal of minimizing secondary injury [13]. As the primary brain injury is often irreversible, care is focused on maintaining adequate cerebral blood flow, oxygenation, and normalization of ICP [14,15]. The guidelines in the treatment and management used by most neurosurgery centers worldwide were set forth by the Brain Trauma Foundation in 2016 with the intent of standardizing management of a severe TBI. These guidelines have been in development for over 25 years and have been associated with a 50% reduction in mortality and costs of patient care [16,17]. Prehospital and emergency department (ED) care is focused on maintaining airway, breathing, and circulation with the use of endotracheal intubation and hypertonic saline as indicated. In the ED, advanced trauma life support guidelines will be followed, and imaging performed [6].

A neurosurgery consult should be made as soon as the diagnosis of a moderate or severe TBI is made. The surgical intervention is a main pillar of TBI management to reduce ICP and brain swelling and manage intracranial hypertension. The indications for surgery include hematomas, increased ICP refractory to medical management, or evidence of mass effect and midline shift. Indication for surgery is guided by the CT findings and GCS [6,13]. In this review, we examined the role of surgical procedures including hematoma evacuation, decompressive craniotomy, ventriculostomy and cisternostomy for hydrocephalus, surgical interventions for contusions and penetrating traumas, and cranioplasty in TBIs, in relation to patient outcomes and complications.

## Review

Role of surgery in contusions

Cerebral contusions are the most common cause of irreversible damage to brain tissue causing significant long-term deficits and deaths. Road traffic accidents (RTAs) and falls are the two most prevalent etiological causes of contusions which are frequently encountered in frontal and temporal lobes. Primary injury in contusions is caused by the transfer of kinetic energy of the traumatic impact into the brain tissue and the progression is related to bleeding from fractured micro vessels. Hemorrhagic contusions lie in the brain parenchyma and blood is the cause of secondary insult in TBIs resulting in post-traumatic seizures [18]. Magnetic resonance imaging (MRI) performed within the first 72 days of injury reveals a necrotic core, comprising an area of compromised cerebral blood flow surrounded by a peri contusional ischemia, called penumbra, due to vasogenic edema and local thrombocyte aggregation and inflammation [18,19]. Bifrontal contusions present in conscious patients with minimal signs and symptoms, who rapidly worsen and require decompression surgery, due to the close proximity of midline structures and rostro caudal displacement of the brain [20].

Indications for Surgery

Clinical deterioration or radiological findings of the patient can be a standalone indication for surgery. Clinical symptoms rapidly worsen in the first 24 hours of injury, rarely continuing to deteriorate in the next 3-4 days. Many studies state that increasing contusion size is an indicator of deterioration usually assessed by computed tomography (CT). Vulnerable populations in whom the size of contusion and increased complications include those with advanced age, preexisting vascular abnormalities, males, chronic alcoholism, chronic hypertensives, and current smokers. Some studies say that a Glasgow Coma Score of <8 has an increased risk of poor prognosis, use of mannitol >2 weeks and greater loss of blood also have a poor outcome. Contusions due to RTAs tend to have a worse prognosis compared to contusions due to falls. Another common indicator in labs for poor prognosis is the derangement of coagulation status, prothrombin time (PT), and INR lab values of the patients and <150mg/di values of triglycerides [19,21,22].

Contusions larger than 20 ml and contusions in the frontal lobe have 5 times more risk of unfavorable outcomes. The basi-frontal lobe is epileptogenic in nature and has poor prognosis; when patients develop seizures, rapid surgical intervention is needed [18,23]. Contrecoup contusions have more risk compared to coup contusions due to increased volume. Patients with concomitant hemorrhage, cisternal compression, or skull fractures tend to have a 2-3 times worse prognosis [18,19]. The contusion index is a useful modality for determining the prognosis and type of intervention and is calculated as a product of depth of contusion and extent of contusion with scores ranging from 0-9. 1-3 scores correspond to GCS 13-15, and patients show good recovery with conservative management. Scores of 4 to 8 require surgical intervention, whereas a score of 9 corresponds to a 100% poor prognosis despite different types of intervention [21]. On admission, patients with GCS score <13.1 or a contusion volume of >62.9cm3 on day 2 post-injury showed persistently elevated ICP despite mannitol administration, or a 2-point decrease in GCS needed surgical interventions [24]. Supratentorial traumatic lesions and Croup injury were a major cause of cerebellar contusions [25].

Patient Outcomes of Surgery

Carnevale et al. conducted a retrospective study of 726 patients with traumatic cerebral intraparenchymal hemorrhage with a mean age of 50 years with 96% blunt injuries including 66% falls and 30% RTA patients [26]. On average, the volume of contusion expanded by 61.6% (4.76ml) at a rate of 0.71ml per hour. This study found a positive association between hemorrhagic progressive of contusion (HPC) and increasing age, male sex, and variation in blood pressure resulting in poorer outcomes. Another study found the mean systolic blood pressure (SBP) to be higher in patients with HPC. The study found a direct correlation between the initial size of intraparenchymal hemorrhage and expansion rate and concluded it to be the greatest predictor for blossoming of contusions. Patients with poor GCS (3-8), lower absolute platelet count and higher blood alcohol levels had greater expansion rates. The coexistence of subdural hematoma (SDH), epidural hematoma (EDH), and subarachnoid hemorrhage (SAH) is a positive predictor for poor prognosis. Cepada et al. hypothesized the removal of the tamponade effect in decompressive craniectomy (DC) to be the cause of contusion progression, but the study found inconclusive evidence [27]. Kumari et al. conducted a prospective study of 250 patients at a tertiary hospital, 70% of cases were of road traffic accidents, 79% were male patients, and 65% of patients had contrecoup injury who had bifrontal brain contusions. Bifrontal contusions have a poor prognosis if not intervened early, as they worsen in the first three days after the impact. The volume of the contusion was calculated using Di Chiro’s formula = [anteroposterior dimension in cm* mediolateral dimension in cm* superoinferior dimension in cm]/2, CT scans were the imaging modality used to assess and follow up the patients. A total of 58 patients received primary surgical intervention based on CT scans. For patients who were managed conservatively, the indicator for secondary surgical intervention was found to be a contusion volume of >30ml. Indicators of worse prognosis were age >46years, train traffic accidents, GCS<8, sluggish/non-reactive pupillary reflex, contusion volume 26-50ml, bilateral splaying of frontal horns of lateral ventricles, anteroposterior shift of genu of corpus callosum, SAH, deformed third ventricle, fully effaced suprasellar/ perimesencephalic cisterns, occipital bone fractures, and delayed development of intracerebral hematoma. They propose prophylactic surgical intervention as the primary management in patients with a critical contusion volume of 27ml [28].

The management of contusions is a dynamic process that requires close monitoring as the volume of contusions increases in up to 40% of patients [29]. The need for surgical intervention depends on several factors like associated intracranial injuries, including subdural hematoma and perilesional edema [20]. The role and timing of surgery in contusions is still a topic of ongoing discussion and controversy in several countries with each having a different opinion based on clinical experience as demonstrated in surveys conducted by the European Brain Injury Consortium. Some neurosurgeons prefer early surgical intervention due to the mass and toxic effects of contusions on the brain and argue that surgery provides a better prognosis. Some physicians argued that delayed toxic effects of intraparenchymal contusions are the reason for poor prognosis in most patients with TBIs [29]. DC is a useful surgery performed in cases of ICP despite medical management; however, it increases contusion size in 13-58% of patients compared to nil surgical intervention [19]. Compared to DC to rapidly reduce the ICP, controlled decompression to gradually reduce ICP followed by periodic removal of brain contusion tissue showed a better prognosis and had a lesser incidence of developing contralateral epidural hematoma, reducing brainstem shifts, intraoperative encephalitis and post-operative cerebral infarction were less [23]. External decompression and clot evacuation are also conducted for patients with severe cerebellar contusions although the prognosis of the patients depends on supratentorial lesions [25]. Patients with cerebellar contusions are prone to delayed complications and show minimum neurological deficits at the time of admission. Even emergency posterior fossa decompression may not significantly improve the prognosis and may develop intracerebellar massive hemorrhage post-procedure. These patients need intensive care monitoring to appropriately deal with unexpected clinical deterioration [20].

Another study showed that when endoscopy-assisted unilateral cerebral falx incision was performed in patients with bifrontal contusions, it shorted the duration of operation time and hospital stay, it also reduced intra and post-operative complications like blood transfusion volume, olfactory nerve injury, delayed intracerebral hematoma and subfascial and centrencephalic herniation, although GCS post-surgery remained same as the control group that underwent bilateral DC [30]. Alahmadi et al. observed that 19% (of 98 patients) of TBI cases required surgical intervention in their study [31], whereas Narayan et al. and Chang et al. observed a rate of 8.9% (of 56 patients) of decompression craniotomies in their study [32,33]. Peterson and Chestnut observed that 50% of patients in their study with contusion volumes over 30 ml required surgical intervention [34], whereas Ratan et al. observed that in their study 49.1% of patients with bifrontal contusion volumes ranging from 32-94 ml required surgery. Numerous studies predict that the blossoming of contusion size around 3-4 days after the traumatic impact is a significant factor for the worsening of prognosis due to an increase in cerebral edema around the contusion core [20]. A case study by Chen et al. found a 27-year-old male with temporal lobe contusion, new onset bradycardia, hypertension, and irregular respiration indicating increased ICP. Low GCS scores, poor pupillary response, and Cushing’s triad may point toward early uncal herniation independent of midline shift in temporal lobe contusions. The patient underwent immediate craniotomy with subtemporal and subfrontal decompression with anterior temporal lobectomy and recovered with a good prognosis [35].

Li et al. retrospectively studied 136 patients with cerebral contusion and laceration combined with cerebral hernia who underwent DC to create a machine learning model that predicts the factors responsible for poor prognosis, which includes surgical treatment to be one of them (OR=4.943,p=0.002), other factors include GCS<8, bleeding volume>30ml, mannitol use >2weeks and patients on anticoagulants during admission. Based on the results, they recommend conservative treatment over surgery in patients with cerebral contusion and laceration combined with cerebral hernia [22].

Faleiro et al. reviewed seven case reports of patients with cerebral contusions, approximately 35% of cases with blunt head trauma result in cerebral contusions. Nine male patients received supraorbital keyhole or minipterional craniotomy surgery, some patients had coexisting SDH, EDH, or diffuse axonal injury. The use of less invasive techniques in the treatment of intraparenchymal hematomas associated with contusions is proposed as they offer satisfactory prognosis; these techniques reduce intra-op blood loss, reduce operating time and recovery phase, and have a better cosmetic outcome, they suggest the use of small areas of corticectomy ( ~1-2 cm) to reach the core of the contusion and hematoma evacuation can be done through same opening through suction and irrigation, cautery for hemostasis and surgical microscope for better visibility. They suggest that this minimally invasive approach should be offered to patients as an option, particularly in traumatic intraparenchymal hematomas associated with contusions [36]. Flint et al. analyzed 40 patients who underwent unilateral decompressive hemicraniectomy and found post-operative hemorrhagic expansion of contusions in 58% of patients based on CT scans and it was a common occurrence after hemicraniectomy and is associated with increased mortality [37]. Mino et al. performed neuro-endoscopic target aspiration (lesionectomy) of the necrotic core for delayed progression of cerebral contusions in 10 patients (mean age=67 years) in cases where standard decompression craniotomy could not be used. The necrotic core that was aspirated using this technique was serous liquid in nature, and post-surgery good prognosis was seen in patients with reduction in peri-confusional edemas within three days [38]. The results reported were insufficient to predict that neuro-endoscopy is more effective or preferable than normal surgical procedures due to the variability of operative scheduling, age, and consciousness level. Older patients may be especially well-suited for minimally invasive procedures, as they may be less able to withstand the surgical trauma of a traditional craniotomy than younger patients. In the near future, randomized clinical trials should concentrate on assessing this method to ascertain the possible function of neuro-endoscopy in TBI as well as its related consequences [39].

Gregson et al. collected data from 1541 patients from STITCH (TRAUMA) trials and used meta-analysis to stratify data based on GCS scores and spontaneous or traumatic injury. The benefits of surgery in traumatic cause and spontaneous hemorrhage were OR= 0.48, p value=0.14 and OR=0.7, and p value= 0.07 respectively. Patients with GCS 9-12 and a large spontaneous ICH obtain the greatest benefit of early surgery compared to extremes of GCS and traumatic cause [40]. Although surgery did not improve functional outcomes, early contusionectomy significantly improved mortality and Rankin Scale and Glasgow Outcome Scale-Extended. However, the benefits of surgery for moderate-sized contusions and patients in good clinical states remain controversial. Additional studies are needed to better understand this topic [39]. Wei et al. studied 446 patients' frontal contusions in high-altitude regions and found that conservative treatment showed the best healing rates followed by patients who underwent surgery and retained the bone flap. Removal of bone flaps during the surgery was associated with worse outcomes [41]. Zhaofeng et al. did a retrospective analysis of 2510 patients with traumatic bifrontal contusions, 19% of patients underwent bifrontal traumatic craniotomy, and the operation timing was the key determining factor in recovery of patients as traumatic bifrontal contusions deteriorate rapidly resulting in central herniation even in conscious patients (led to the term “talk and die”), with early surgical intervention 81% patients with GCS 13-15 recovered well [42]. Janzen et al. found two cases of young males developing a rare complication of syndrome of trephined months after undergoing bifrontal decompressive craniectomy which gravely affects the rehabilitation of patients, and cranioplasty was shown to improve the outcome [43]. All studies are summarized in Table 1.

**Table 1:** Summary of studies for surgery in contusions DC: Decompressive Craniectomy, ICP: Intracranial Pressure, EDH: Epidural Hematoma, SDH: Subdural Hematoma, GCS: Glasgow Coma Scale.

Author name	Surgical procedure	Indications	Patient outcomes
Adatia et al. [19].	Decompressive craniectomy (DC)	Increased ICP refractive to medical management	Although the benefits of surgery outweigh the risks in patients with poor prognostic factors, DC is being replaced by newer minimally invasive techniques. The risk of increased contusion volume in 13-58% of patients, increasing disability rates.
Chen et al. [23].	Controlled decompression	Severe traumatic brain injury	Better prognosis and reduced post-operative complications in patients compared to DC
Sato et al. [25].	External decompression and clot evacuation	Traumatic cerebellar contusions prone to rapid deterioration	Supratentorial lesions and delayed intracerebellar hemorrhages decide patient prognosis
Dong et al. [29].	Endoscopic-assisted unilateral cerebral falx incision	Dissymmetric bilateral frontal contusions	Minimally invasive surgery reduced operation time, hospital stay, inter/post-op complications like olfactory nerve injury, delayed intracerebral hematoma, subfalcine and centrencephalic herniation.
Chen et al. [35]	Craniotomy with sub temporal and sub frontal decompression with anterior temporal lobectomy	Temporal lobe contusion with positive crushing's triad	This study found that the patient had a good outcome with minimal disability and behavioral change after surgery despite poor preoperative prognostic factors.
Li et al. [22].	Decompressive craniotomy	Cerebral contusion and laceration combined with cerebral hernia	This study found that conservative management had better patient outcomes than surgery in this type of injury.
Faleiro et al. [36].	Supraorbital keyhole or mini-pterional craniotomy	Cerebral contusions+ SDH, EDH or diffuse axonal injury	These minimally invasive techniques have better patient outcomes in cases of traumatic intraparenchymal hematomas associated with contusions, hence a viable surgical option.
Flint et al. [37].	Unilateral decompressive hemicraniectomy	Severe traumatic cerebral contusion	This type of surgery is associated with increased post-operative hemorrhagic expansion of contusion and increased mortality rate and is not a preferred option.
Mino et al. [38].	Neuro-endoscopic target aspiration of necrotic core of contusion (lesionectomy)	In cases where there was delayed hemorrhagic progression of cerebral contusions and DC could not be used	Good patient outcomes due to surgery, these minimally invasive techniques are especially suited for patients with older age who have poor prognosis with standard surgical procedures.
Wei et al. [41].	Decompressive craniectomy+ removal of bone flap	Frontal contusions in high altitude regions (>2500m elevation)	Conservative management showed the best healing rates at high altitudes followed by patients who underwent surgery and retained the bone flap.
Zhaofeng et al. [42].	Bifrontal decompression craniotomy	Traumatic bifrontal contusions	Early surgical intervention in patients with GCS 13-15 had an 81% recovery rate.

Hematoma evacuation

A hematoma is a localized collection of blood outside the blood vessels, typically resulting from hemorrhage or trauma. Intracranial hematomas are particularly dangerous due to the limited capacity of the intracranial cavity to accommodate sudden increases in ICP. The expansive force of a rapidly enlarging hematoma can reduce cerebral perfusion pressure, compromising blood flow through cerebral vessels and resulting in ischemia [44,45]. In order to discuss the role and relevance of hematoma evacuation in the treatment of traumatic brain injury, it is imperative to classify and understand the different types of intracranial hematomas.

Indications for Surgery

An EDH occurs when blood accumulates between the dura mater and the skull, typically due to a tear in the middle meningeal artery or vein, often following a skull fracture [46]. EDH accounts for 2-3% of all head injuries and is more common in young adults, particularly males, due to their higher likelihood of sustaining trauma [47]. Indications for surgical evacuation of EDH include a hematoma volume greater than 30 mL, a midline shift greater than 5 mm, a GCS score less than 8, and pupillary abnormalities [46]. Early intervention is critical to prevent irreversible brain damage and improve outcomes.

SDH involves the accumulation of blood between the dura mater and the arachnoid mater, usually resulting from the tearing of bridging veins [46]. SDH can present acutely, subacutely, or chronically, with acute SDH being more severe due to rapid blood accumulation. Acute SDH accounts for approximately 11% of all TBIs and is associated with a high mortality rate [48,49]. Surgical intervention for acute SDH is typically indicated when the hematoma thickness exceeds 10 mm or the midline shift is greater than 5 mm. Other indications include a declining neurological status and signs of elevated ICP. For chronic SDH, surgery may be required if there is a progressive neurological decline or a significant mass effect. If pursued, minimally invasive techniques have shown more favorable outcomes [46,50].

Intracerebral hematoma/hemorrhage (ICH) involves bleeding within the brain parenchyma, often due to trauma or rupture of a blood vessel [47]. ICH accounts for 8-13% of all stroke cases and has a high morbidity and mortality rate, particularly in the elderly [51]. Surgery for ICH is considered for large hematomas with significant mass effect, deteriorating neurological status, and elevated ICP. Basal cistern status, coma score, and the severity of edema surrounding the intracerebral hematoma should also be used, in addition to ICP monitoring, to help guide the management of patients with traumatic intracerebral hematoma [52]. The location of the hematoma also plays a crucial role in the decision-making process, with lobar hemorrhages being more amenable to surgical intervention compared to deep-seated hemorrhages [48]. Indications for intracranial hematomas are summarized in Table 2.

**Table 2:** Indications for intracranial hematomas GCS: Glasgow Coma Scale, ICP: Intracranial Pressure

Hematoma type	Epidural hematoma	Subdural hematoma	Intracerebral hematoma
Indications [46-52]	Hematoma volume greater than 30 mL, a midline shift > 5 mm, a Glasgow Coma Scale (GCS) score less than 8, and pupillary abnormalities.	Acute SDH: hematoma thickness > 10 mm, midline shift > 5 mm, elevated ICP or declining neurological status.	Significant mass effect, deteriorating neurological status, and elevated ICP. Basal cistern status, coma score, and the severity of edema surrounding the intracerebral hematoma and location are important to be considered.

Types of Surgery

Craniotomy involves the surgical removal of a section of the skull, known as a bone flap, to access and evacuate the hematoma directly. This technique is primarily indicated for large epidural and acute subdural hematomas where rapid decompression is necessary to prevent brain herniation and reduce ICP [46]. The procedure allows for thorough evacuation of the hematoma, control of bleeding sources, and repair of the dura mater. Post-operatively, patients are closely monitored in the intensive care unit (ICU) for complications such as rebleeding, infection, edema and seizures. Craniotomy is associated with significant morbidity, mortality and hospitalization costs; however, timely intervention can result in favorable outcomes, particularly in patients with good preoperative neurological status [47,53].

Burr hole evacuation involves drilling one or more small holes into the skull to allow for drainage of the hematoma. This technique is particularly useful for chronic subdural hematomas, which develop over weeks to months and often present with progressive neurological decline [48]. The procedure is less invasive than craniotomy and can often be performed under local anesthesia, making it suitable for elderly patients and poor surgical candidates with significant comorbidities. After drilling the burr holes, a catheter is inserted to irrigate and drain the hematoma. Post-operative care focuses on monitoring for reaccumulating of the hematoma and ensuring adequate brain re-expansion. Burr hole evacuation has a high success rate, with lower complication rates and shorter recovery times compared to craniotomy [53].

Endoscopic evacuation utilizes small incisions and an endoscope, a flexible tube with a camera and light at the end to visualize and evacuate the hematoma. This minimally invasive technique is gaining popularity due to its ability to reduce surgical trauma, minimize brain tissue manipulation, and shorten recovery times [51]. The procedure involves inserting the endoscope through a small cranial opening to locate the hematoma and using specialized instruments to aspirate and remove the blood. Endoscopic techniques are particularly advantageous in cases where the hematoma is located in eloquent brain areas or in deep-seated regions that are difficult to access with traditional methods without causing further injury to brain tissue. The reduced invasiveness of endoscopic evacuation translates to lower risks of infection, less post-operative pain, and faster rehabilitation [53].

**Figure 1:**
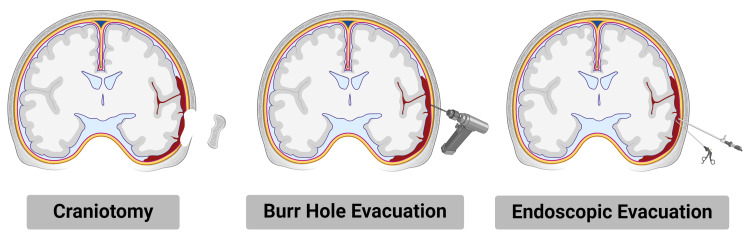
Types of surgeries to evacuate intracranial hemorrhages The figure was made using biorender.com

Stereotactic aspiration involves using imaging guidance, such as CT or MRI, to precisely place a catheter for hematoma aspiration. This technique is often employed for deep-seated intracerebral hematomas, regions where direct surgical approaches pose significant risks. The stereotactic frame or a frameless navigation system is used to calculate the optimal trajectory for catheter placement, ensuring minimal disruption to surrounding brain tissue. Once the catheter is in place, the hematoma is aspirated, and the catheter may be left in situ for continued drainage if necessary. Stereotactic aspiration offers the advantage of precision, reducing the risk of collateral damage and improving outcomes in patients with challenging hematoma locations. This technique is particularly useful in patients who are not ideal candidates for open surgery due to medical comorbidities or the hematoma's location (Figure 1) [51-54].

Patient outcomes and prognostic factors

The outcomes of hematoma evacuation are dependent on a number of prognostic factors including the type and size of the hematoma, the patient’s initial neurological status, the timing of surgery since initial presentation, and the presence of comorbidities. Outcomes for EDHs are quite favorable with early surgical intervention. Many patients are able to make a full recovery, particularly when the initial GCS score is greater than 8 and there are no pupillary abnormalities at presentation [46].

The outcomes for acute SDHs, unlike EDH, are typically much worse due to the rapid accumulation of blood and concomitant brain injury. In a study of patients ≥70 years of age operated on for post-traumatic acute SDH (ASDH) in a three-year period in five Italian hospitals patients were divided into three surgical timing groups from hospital arrival: ultra-early (within 6 h); early (6-24 h); and delayed (after 24 h). The outcome was measured at discharge using two endpoints: survival (alive/dead) and functional outcome at the Glasgow Outcome Scale (GOS). Results included 136 patients. About 33% died as a result of the consequences of ASDH and among the survivors, only 24% were in good functional outcome at discharge. Surgical timing groups appeared different according to presenting the GCS, which was on average lower in the ultra-early surgery group, and radiological findings, which appeared worse in the same group. Delayed surgery was more frequent in patients with subacute clinical deterioration. Surgical timing appeared to be neither associated with survival nor with functional outcome, also after stratification for preoperative GCS. Overall, 91 patients (67%) were alive at discharge, but only 33 (24% of the total number of patients) appeared to have a good functional outcome [53].

Factors with a specific predictive value for SDH include the patient’s age, hematoma thickness, midline shift and initial GCS score, with both elderly patients and patients presenting with severe neurological deficits having poorer outcomes [48]. A treatment strategy preferring an aggressive approach of acute surgical evacuation over initial conservative treatment was not associated with better functional outcome. Outcomes following surgical evacuation of ASDH in patients aged 60 years and above are poor [55]. Therefore, in a patient with acute subdural hematoma for whom a neurosurgeon sees no clear superiority for acute surgery, initial conservative treatment might be considered [56]. Chronic SDHs, on the other hand, often have a benign prognosis with good turnout, particularly with burr hole evacuation. Burr-hole craniostomy was found to be associated with lower recurrence rates, when compared to other surgical procedures. They also have lower risk of complications, particularly if managed promptly [53]. Recurrence rates after surgical evacuation range from 5 to 30%. Factors predicting recurrence remain debated and unclear [57].

For ICH, the prognosis is highly dependent on the location and volume of the hematoma, as well as patient age and comorbidities, and initial GCS score. Deep-seated hematomas with higher volumes are associated with a worse prognosis [51]. Early and accurate intervention, particularly with minimally invasive techniques such as stereotactic aspiration and endoscopic evacuation, are associated with better outcomes due to reduced surgical trauma and the preservation of surrounding brain tissue [47]. The effectiveness of hematoma evacuation is contingent on a rapid diagnosis, timely surgical intervention, comprehensive post-operative care of secondary brain injuries and rehabilitation for optimal functional recovery [58]. Table 3 summarizes the surgical outcomes as per procedure.

**Table 3:** Summary of patient outcomes as per surgery in intracranial hematoma

Procedure name	Indication for surgery	Patient outcomes
Craniotomy [46,47,53]	Large epidural and acute subdural hematomas where rapid decompression is necessary to prevent brain herniation and reduce intracranial pressure (ICP).	Favorable outcomes, particularly in patients with good pre-operative neurological status.
Burr hole evacuation [48,53]	Chronic subdural hematomas, which develop over weeks to months and often present with progressive neurological decline.	Burr-hole evacuation was found to be associated with lower recurrence rates, when compared to other surgical procedures. It has a high success rate, with lower complication rates and shorter recovery times compared to craniotomy.
Endoscopic evacuation [51,53]	Endoscopic techniques are particularly advantageous in cases where the hematoma is located in eloquent brain areas or in deep-seated regions that are difficult to access with traditional methods without causing further injury to brain tissue.	The reduced invasiveness of endoscopic evacuation translates to lower risks of infection, less post-operative pain, and faster rehabilitation.
Stereotactic aspiration [51-53]	This technique is often employed for deep-seated intracerebral hematomas, in which direct surgical approaches pose significant risks.	Early and accurate intervention, particularly with minimally invasive techniques such as stereotactic aspiration and endoscopic evacuation, are associated with better outcomes due to reduced surgical trauma and the preservation of surrounding brain tissue.

Decompressive craniectomy for elevated ICP in traumatic brain injuries

Indications and Guidelines

**Figure 2:**
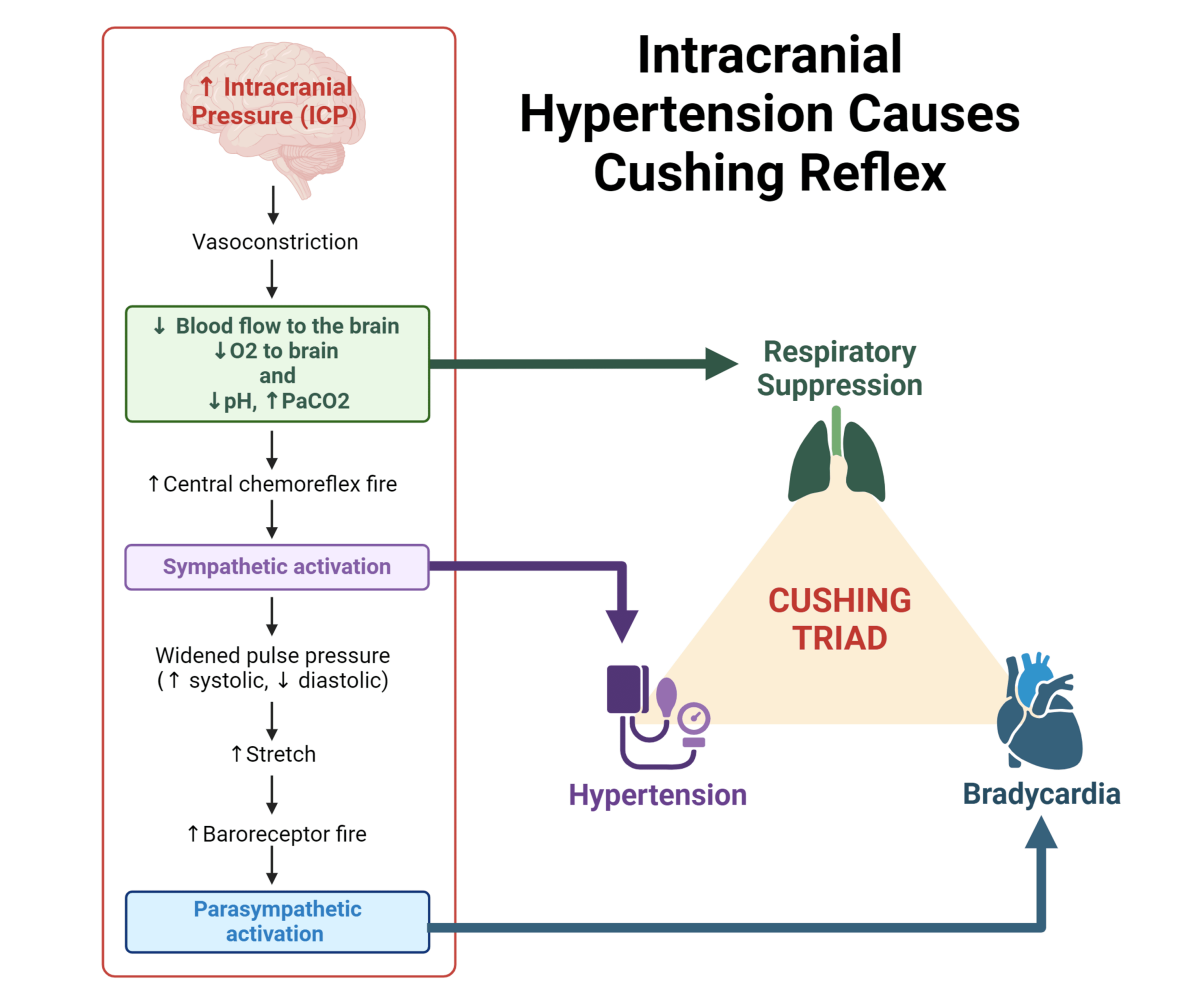
Elevated intracranial pressure leads to Cushing's triad The figure was made by authors using biorender.com

**Figure 3:**
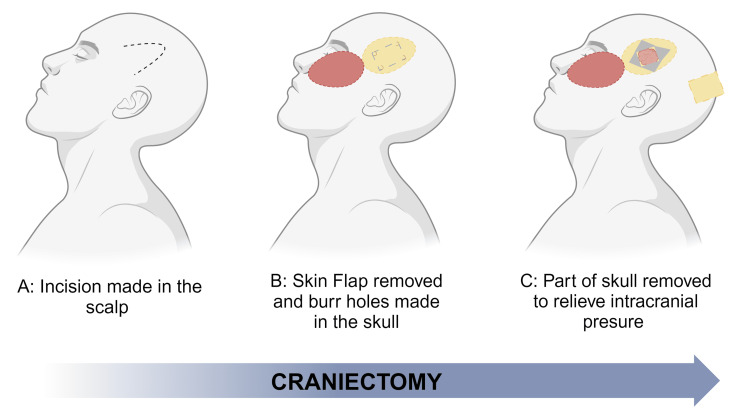
Craniectomy The figure was made by authors using biorender.com

ICP elevation is a common symptom in a severe TBI. The significant increase in intracranial substances can lead to compartment syndrome, which obstructs blood flow to the brain and can cause brain herniation, death, or disability. Increased ICP can lead to Cushing’s triad comprised of bradycardia, respiratory difficulty and hypertension (Figure 2) [59,60]. DC is a surgical approach to reducing ICP. This procedure involves cutting through the dura mater and removing part of the cranium to prevent secondary parenchymal damage or brain herniation (Figure 3) [61]. There are two classifications of decompressive craniectomy: primary and secondary. Primary decompressive craniectomy occurs when the removed part of the skull is not immediately replaced due to brain swelling or anticipated swelling after the procedure, especially when an intracranial mass lesion is removed soon after a head injury. Secondary DC refers to the removal of a portion of the skull later in the treatment plan to reduce ICP after other treatment options have failed [60,62].

Hawryluk et al. highlighted the Brain Trauma Foundation’s Guidelines for the management of severe traumatic brain injury, providing several recommendations regarding secondary DC. Secondary DC is beneficial for refractory ICP elevation and is advised to improve survival rates and positive outcomes. However, secondary DC performed for early refractory ICP elevation is not recommended for enhancing survival rates and outcomes. A large frontotemporoparietal DC (at least 12 × 15 cm or 15 cm in diameter) is recommended over a small frontotemporoparietal DC to lower mortality and improve neurological outcomes in patients with severe TBIs. Furthermore, secondary DC performed for either early or late refractory ICP elevation is suggested to reduce ICP and the duration of intensive care, though its relationship with favorable outcomes remains uncertain [63].

Although primary DC is widely used as a surgical approach to TBI, there are inconsistent findings regarding its effectiveness in reducing ICP. Studies suggest that secondary DC may promote recovery for refractory ICP elevation while providing no benefit when used as a primary approach. Additionally, in countries with limited hospital resources, primary DC is used more frequently than secondary, possibly due to resource constraints like available beds and space, though this has not been confirmed [64,65].

Surgery for TBIs is determined by the patient's neurological functioning level, the size of a hematoma, or the size and severity of an intracranial mass. Vitali et al. gave a clear overview of the indications of DC and its techniques [61]. DC techniques vary based on different scenarios. There are two main classifications: infratentorial or supratentorial DC. Infratentorial or suboccipital DC is mainly used for non-severe TBIs due to the rare involvement of the posterior fossa in severe TBIs and is primarily used for ischemic or hemorrhagic posterior fossa strokes. Supratentorial DC is further divided into unilateral or bilateral approaches. Each technique has its own indications. Bilateral DC includes bifrontal craniectomy and bilateral frontotemporal craniectomy, aiming to decompress both hemispheres in cases of diffuse brain edema without localized lesions. The primary indication for bifrontal DC is severe TBIs with frontal contusions and diffuse brain edema, though recent guidelines do not recommend its use as it does not improve long-term outcomes despite controlling ICP. Unilateral DC is the most common technique and involves a fronto-temporo-parietal craniectomy. The patient is positioned supine with the head turned to the contralateral side, and a large question mark-shaped incision is made, taking care to preserve the superficial temporal artery to avoid ischemic complications. A fronto-parieto-temporal craniectomy is then performed, which should be no smaller than 12 × 15 cm or 15 cm in diameter and should extend toward the floor of the temporal fossa to provide adequate decompression. Small decompressions can be inadequate and may cause further brain damage by compressing the brain cortex and cortical veins, enhancing brain herniation [60].

Patient Outcomes and Complications in DC

A trial conducted by Beck et al. assessed decompressive craniectomy along with medical treatment versus medical treatment alone for patients with severe deep supratentorial intracerebral hemorrhage. This randomized trial included adults from 42 stroke centers across several European countries. The primary outcome measured was a score on the modified Rankin Scale at 180 days. The study found that 44% of participants in the decompressive craniectomy group had unfavorable outcomes, compared to 58% in the medical treatment group. However, the trial was stopped early due to a lack of funding, limiting the robustness of its findings [66].

A study conducted by Kolias et al. analyzed patients with traumatic intracranial hypertension treated with decompressive craniectomy versus standard medical care were analyzed. The study was conducted across 52 centers in 20 countries. There were 408 patients who were involved and evaluated according to their outcomes after 24 months. Results showed that patients in the surgical group had reduced mortality and higher rates of vegetative state and severe disability compared to the medical group. Although the surgical intervention increased life expectancy, it also led to higher rates of severe disability, giving evidence to a thin line between survival and quality of life [67].

Hutchinson et al. conducted a trial that focused on the six-month outcomes for patients with TBIs and resistant intracranial hypertension. This study was implemented through what is known as a Randomized Evaluation of Surgery with Craniectomy for Uncontrollable Elevation of Intracranial Pressure (RESCUEicp) to evaluate the outcome of a decompressive craniectomy as a secondary approach to TBI and/or ICP hypertension compared to only medical care. The cohort consisted of patients between the ages of 10 and 65 years that presented with TBI, and an unusual CT brain scan with ICP above 25 mm Hg for 1 to 12 hours. Three hundred and eighty-nine patients were analyzed for the primary outcome of six months (201 patients in the surgical group and 188 in the medical group), and 373 were analyzed for the secondary outcome of 12 months (194 in the surgical group, and 179 in the medical group). Results of the study at six months showed the following results of the surgical group compared to the medical group: 8.5% compared to 2.1% in a vegetative state, 21.9% compared to 14.4% resulted in mild disability, 15.4% compared to 8% with severe disability, 23.4% compared to 19.7% resulted in moderate disability and favorable recovery was 4% compared to 6.9%. At 12 months the following was shown: 6.2% compared to 1.7% in a vegetative state, 18% compared to 14% with mild disability, 13.4% compared to 3.9% with severe disability, 22.2% compared to 20.1% resulted in moderate disability and 9.8% compared to 8.4% resulted in a favorable recovery. Overall, it was found that decompressive craniectomy significantly reduced death rates compared to medical care alone. However, it also resulted in higher rates of vegetative state and severe disability. Despite these adverse outcomes, the rates of moderate disability and good recovery were similar between the surgical and medical groups, highlighting the variety in the results of surgical intervention [68].

For a subset of individuals with severe high-energy injuries, DC was suggested to be useful. Patient recovery was found to be improving over time in those patients treated with DC [69]. Compared to patients in the medical group, those in the surgical group had a higher chance of improving over time. At 24 months, there were 21 more survivors for every 100 patients treated with surgical rather than medical management [63]. A statistically significant drop in ICP readings was found among the DC group in contrast to the control groups. With a pooled risk ratio of 0.67 (95% CI: 0.51-0.89), the DC group appeared to have had a statistically significant 31% reduction in death rates [70]. Similarly, a study by Pingue et al., suggested that after monitoring their patients for six months post-DC in their rehabilitation program, although the risk of unstable seizures was high in those patients who underwent DC, there were no significant associations between DC and mortality rate or poor functional outcome [71].

On the other hand, a previous study expressed that in 155 adults with severe diffuse TBI, bi-front temporoparietal DC decreased the duration and number of interventions needed for the management of intracranial hypertension and days in the ICU compared to standard care. However, in the DECRA trial conducted, the mortality rate and unfavorable outcomes were higher for the DC group compared to the standard care at 12 months post-intervention. In total, 17% of patients in the standard care group and 37% of patients in the craniectomy group experienced one or more surgical or medical problems. The key outcome (functional evaluation on the Extended Glasgow Outcome Scale) was poorer in the craniectomy six months after the injury. The odds ratio in the craniectomy group for a worse functional outcome was 1.84 (95% confidence interval [CI] = 1.05 to 3.24; P=0.03) [72]. In comparison to craniotomy, more DC patients experienced hospital readmissions at some time throughout the one-year (39% vs. 25%) and two-year (48% vs. 35%) follow-up periods. In one year, more DC patients had been admitted to the hospital for seizures, neurological conditions, infections, orthopedic surgeries, and other general health issues [73].

Several complications are anticipated during and post-DC. Based on the timing of the complications, they can be divided as early (within four weeks) and late or delayed (more than four weeks) complications. Early complications include hemorrhage, external cerebral herniation, wound complications like dehiscence due to ulcerations or necrosis, cerebrospinal fluid leakage or fistula formation, post-op infections, and seizures or epilepsy. The delayed complications include subdural hygroma, hydrocephalus, and syndrome of the trephined [70]. Among these, the most frequent complications were those associated with CSF disturbances which attributed to 18.4% of the frequency of complications, followed by hemorrhagic (11.9%) and infectious or inflammatory (5.5%) [74].

In the most recent review by Munch et al., they implied that in a randomized clinical trial with 408 patients, the DC group showed improved survival and a lower death rate six months following traumatic brain injury as compared to the standard care group [75]. Reduced midline movement of brain tissue and enhanced mesencephalic cistern visualization are two additional advantages of DC for TBI patients [64]. Consequently, when tested six months after the injury, younger (i.e. less than 50 years old) TBI patients who had undergone DC showed superior GOS scores than older patients. Additionally, it was found that in TBI patients, an early DC (4.5 ± 3.8 h after TBI) was more advantageous than a delayed DC (56.2 ± 57.0 h) [76].

When compared to control groups, DC can be regarded as an essential surgical intervention for lowering death rates among TBI patients although they do have higher rates of complications and rehospitalizations. Additional randomized clinical trials are necessary in light of contradictory results about the effects of acute and late complications and on quality of life. Some major trials and studies are summarized in Table 4.

**Table 4:** Summary of major trials and studies for decompressive craniectomy DC: Decompressive Craniectomy

Author name	Trial name	Study arms	Outcomes
Beck et al. [66]	SWITCH	DC + medical treatment vs medical treatment alone	14% decline was reported in unfavorable outcomes among the patients who underwent surgery as compared to patients who received only medical treatment.
Kolias et al. [67]	Secondary analysis of RECUEicp Trial	DC vs medical therapy alone	Surgery reduced death rate although disability and paralysis significantly increased among patients who underwent surgery.
Hutchinson et al. [68]	RESCUEicp	DC vs medical therapy alone	DC improved mortality rates at 12 months follow-up. However, severe disabilities and vegetative state were significantly greater.
Pingue et al. [71]	-	DC vs medical therapy alone	DC was related to occurrence of unprovoked seizure. However, no correlation was established between DC and unfavorable outcomes
Cooper et al. [72]	DECRA	DC vs standard therapy	Duration of length of stay in ICU and need for re-operations were lower in DC group. However, death rate and poor outcomes were markedly higher
Kelly et al. [73]	-	DC vs craniotomy	Post-surgical complications and need for re-admission was higher in DC group as opposed to craniotomy group.

External ventricular drain (EVD), ventriculostomy and cisternostomy

TBIs remain the leading cause of death mainly because of increased ICP. ICP-guided therapy can be performed via intraparenchymal fiberoptic monitor (IPM) or EVD. Use of EVD is considered the gold standard for measuring ICP while allowing CSF drainage. In contrast, IPM is considered in the absence of hydrocephalus because of lower risk of hemorrhage and infection than EVD. The advantage of the ventricular monitoring device is that it can be utilized to outflow excess CSF in cases of a sustained increase in ICP, which is defined as pressure greater than or equal to 20 mmHg for 5 minutes or more, while the disadvantage is that simultaneous monitoring as well as CSF drainage is not possible [77].

Currently, there are no standard guidelines for commencing ventricular drainage for elevated ICP. A study proposed ICP levels of above 15 mmHg as the triggering point for initiating drainage using ventriculostomy as a primary procedure. Others recommended a threshold of 20 mmHg, with variable duration ranging from 5 to 10 mins. Some surgeons have also implemented CSF drainage as a last resort with ICP above 25 mmHg, with no resolution following DC [78].

Mouchtouris et al. analyzed 2305 patients who suffered TBIs and underwent IPM and EVD placement. 32.2% of IPM and 26.6% of EVD patients died during the hospital stay. IPM was a negative predictor with a higher mortality rate with an odd ratio of 1.40. Score-adjusted analysis showed a 28% reduction in the death rate and 14% reduction in the duration of hospital stay among patients with EVD [79]. A systemic review of 21 studies conducted by Chau et al. reports total hospital mortality varies from 5 to 30% among patients with TBIs with EVD placement. All of these studies included patients in the early EVD groups, classified as EVD placed concurrently with IPM. Patients were evaluated at 3 months, 6 months, an average of 8.7 months, and 12 months after injury. Mortality at three months or later post-injury ranges from 9 to 35%, while 34-67% of patients had an unfavorable outcome. Seven studies, including 689 patients with an EVD, reported the number of patients requiring DC for refractory intracranial hypertension. The early EVD group had 0-55% of patients requiring DC, while for the late group, the frequency was 12%. Six studies reported length of stay in the intensive care unit (ICU) with a mean ranging from 8.94 to 20.1 days [80].

**Figure 4:**
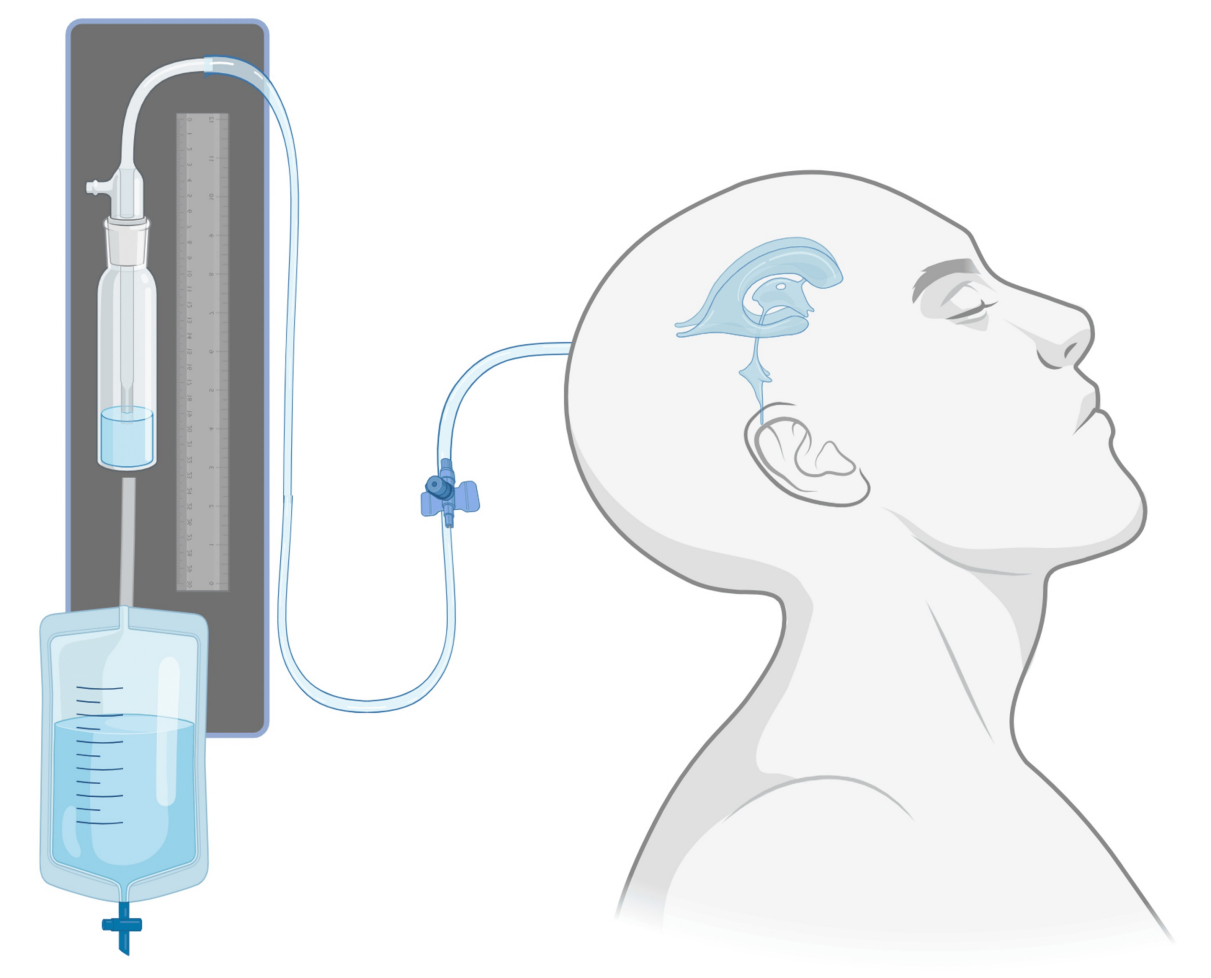
External ventricular drain (EVD) The figure was made using biorender.com

Encarnacion et al. conducted a study in which 24 patients (80%) underwent DC + cisternostomy, whereas six patients (20%) underwent cisternostomy exclusively [81]. The mortality rate was 12.5% (three patients) and 16.7% (one case) in the DC + cisternostomy group and in the cisternostomy alone group, respectively. The overall mortality was 13.3%, with a morbidity rate of 50% in patients with initial GCS scores of 4 and a mortality rate of 16.7% in patients with GCS scores of 6. Giammattei et al. compared outcomes among 40 patients with TBIs who underwent adjuvant cisternostomy (AC) and DC [82]. Patients in the AC cohort needed mechanical ventilation and ICU admission for a lesser duration and were discharged with superior GCS scores. AC patients had overall superior positive outcomes at six months follow-up and lower post-procedure ICP levels. Findings are summarized in Table 5 (Figure 4).

**Table 5:** Summary of findings in external ventricular drain (EVD), ventriculostomy, and cisternostomy DC: Decompressive Craniectomy

Procedure	Indications	Patient outcomes
External ventricular drain (EVD) placement, ventriculostomy and cisternostomy [77-81].	Drainage of CSF in elevated ICP 15 to 20 mmHg for 5 to 10 mins.	ICU admission and length of stay significantly reduced. Mortality rate decreased. Need for decompressive craniectomy was markedly lower among patient with late EVD placement. Need for mechanical ventilation was lower among patients who underwent cisternostomy along with DC.

Penetrating injury

Penetrating brain injuries (PBIs) represent a critical subset of TBIs, where foreign objects breach the cranial vault and directly impact brain tissue. Missile injuries, such as those caused by bullets, are typically more severe due to the high velocity and energy transfer, leading to extensive brain damage. Non-missile injuries, including stab wounds and injuries from sharp objects like nail guns, tend to cause localized damage but can still be life-threatening depending on the affected brain areas. Penetrating injuries involving the orbit or cranial bones pose unique challenges due to their proximity to critical structures like optic nerves and major blood vessels. Advanced imaging techniques help establish early and accurate diagnosis which helps to predict outcomes of the injuries. Initial assessment plays an important role in determining the severity of the injuries and helps to lay a plan for the management of the patient. Timely surgical intervention, typically within hours of injury, is crucial for mitigating secondary brain damage and optimizing functional recovery. Key objectives in early management include rapid hematoma evacuation, tissue debridement, and vascular injury repair. Prompt surgical and medical management helps prevent complications like increased ICP and cerebral ischemia [83-85].

Surgical intervention is extremely crucial in managing PBIs to prevent further brain damage and infections, control ICP not responsive to medical management, remove injury-causing objects and manage open skull fractures. Early surgical intervention can drastically improve outcomes by addressing complications such as hematomas and brain swelling. Advances in recent times for surgical techniques and neurocritical care have significantly shaped the journey of treatment paradigms, enhancing survival rates and functional recovery. The outcome varies depending upon the context of injury, timely surgical management, accessibility to specialized trauma centers, and rapid response teams. While conservative management and medical therapy suffice for some cases, severe PBIs often necessitate surgical exploration to achieve decompression, hemostasis, and tissue preservation. The choice between operative and non-operative approaches hinges on injury characteristics and patient’s neurological status [86-88].

PBIs in military settings are associated with a death rate of 18.0% overall and a neurosurgical intervention rate of 64.3% of patients, according to a systematic review and meta-analysis of surgical therapy and sequelae [83]. In order to remove the foreign object, debride necrotic tissue, drain hematomas, and repair dural abnormalities, surgical therapy typically involves a craniotomy. For stable patients or lesions near the cranial base, minimally invasive methods like endoscopic removal may be used. Endoscopic-assisted procedures offer minimally invasive options for specific PBIs, facilitating precise visualization and targeted interventions in challenging anatomical sites. These techniques minimize complications, promote faster recovery, and are particularly beneficial for managing injuries involving complex cranial structures [89,90]. For patients with abnormalities on a CT scan and severe traumatic brain injury (GCS ≤8), external ventricular drainage is advised to enable therapeutic CSF draining and ICP monitoring. Treatment should be guided by ICP monitoring and should begin when ICP rises above 20 mmHg for a duration of more than five minutes [91]. External carotid artery ligation, carotid endarterectomy, bypass surgery, direct vascular suturing, and aneurysm cutting or trapping were among the surgical techniques used. Seven patients had hemorrhagic lesions embolized with Gel foam or coils during endovascular operations, and one patient had an ischemic lesion stent placed [92]. The use of a multidisciplinary team comprising neurosurgery, ophthalmology, and interventional neuroradiology and preoperative vascular imaging to assess vascular injury and careful surgical removal of the foreign body to prevent further harm are important aspects of surgical management and complications. In addition, CSF leaks were avoided by repairing the skull base and dural abnormalities, and the risk of infection was decreased by prophylactic antibiotic use [93]. Patients who experience CSF leaks that last longer than 10 to 14 days or who experience problems including uncontrollably developing meningitis or progressive pneumocephalus should consider surgery. In this study, 18% of patients with chronic CSF leaks needed surgical repair because their prolonged rhinorrhea did not resolve after a period of monitoring and conservative therapies [94]. Compared to the non-surgical group, the surgical group saw a noticeably higher infection rate [83]. Seizures occur in 13.2% of cases of central nervous system infections, whereas the complication rate was 13.8%. Furthermore, 5.4% of surgical cases reported fistulas or leaks of CSF fluid [82,93,94]. Rebleeding, severe cerebral edema, and septic shock were among the complications in some of the instances reported [95]. Findings are summarized in Table 6.

**Table 6:** Summary of surgeries in penetrating trauma

Procedure name	Indication for surgery	Patient outcomes
Craniotomy [85-88]	Remove foreign objects, debride necrotic tissue, drain hematomas, repair dural defects	Improved outcomes with timely intervention, but risk of complications like infections and CSF leaks
Endoscopic removal [89,90]	Stable patients or lesions near skull base	Generally good outcomes when performed promptly
External ventricular drainage [91]	Severe TBI (GCS ≤8) with CT abnormalities	Allows ICP monitoring and CSF drainage
Vascular procedures (e.g. embolization, stenting) [92]	Vascular injuries	Can address hemorrhagic and ischemic lesions
Skull base/dural repair [84,94]	CSF leaks lasting >10-14 days	Prevents meningitis and pneumocephalus

A combination of timely surgical intervention, effective infection control, and comprehensive rehabilitation is necessary for the Successful management of PBIs. Future research on the management of TBIs should focus on optimizing the surgical techniques, improving diagnostic protocols, and developing better rehabilitation strategies to enhance outcomes for the patients.

Cranioplasty

Cranioplasty (CP), defined as the reconstruction of cranial defects, not only offers protective effects with an aesthetically pleasing outcome but also reverses the altered physiology post-craniotomy and craniectomy [96]. CP primarily intends to maintain cerebral protection, reconstruct aesthetic appearance and may alleviate cognitive and functional deficits by reinstating the regular cerebrospinal fluid dynamics and improving brain perfusion and thus enhance neurological recovery after TBI [97]. CP is technically demanding and demands certain levels of operator skill levels [98]. CP has been primarily indicated to repair cranial defects after DC of TBI patients and this improves cognitive, functional, and neuropsychological outcomes [97,99,100].

Large defects in adults can be reconstructed with titanium mesh and polymethylmethacrylate overlay [101]. Autologous bone grafts have been considered the gold standard for CP though this has been associated with specific disadvantages, including bone resorption, complications associated with harvesting as well as molding into the defect shape [102]. The timing of CP has been found to be debatable. Though some studies emphasize that early CP reduces the operating time and improves outcomes, the timing of CP depends largely on the indication for craniectomy [102-106]. Immediate CP may be performed for craniectomy for neoplastic invasion of cranium and delayed CP is usually indicated for removal of bone flap for intracranial infection or medically refractory intracranial hypertension. Early CP can be associated with the development of hydrocephalus so this prompts close post-operative monitoring for hydrocephalus symptoms [107].

**Figure 5:**
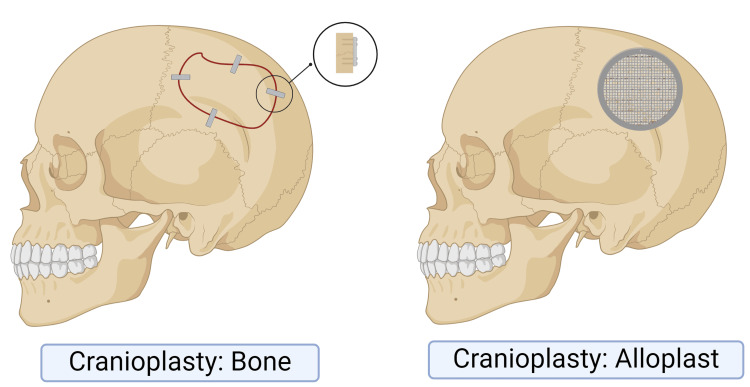
Cranioplasty The figure was made using biorender.com

Though technically straightforward and while a subsequent CP after DC is crucial to prevent sinking skin flap syndrome (SSFP) and to restore cranial integrity, this procedure may lead to many non-trivial complications [108,109]. Previous infection history, immune status, uncontrolled diabetes, type of implant material used, and the technique used to fix the implant are the primary risk factors in CP [102]. CP after TBIs was associated with increased odds of bone flap resorption, infection rates and other complications when analyzed separately and this awareness helps promote the implementation of new strategies to prevent these complications [108]. Post-operative Infections after TBIs can be a significant concern especially among diabetic patients undergoing CP which could be dealt with by mitigation strategies like tighter blood sugar control with sufficient nutrition and adequate use of antibiotics [110]. The rate of treatment failure was less when an immediate single-stage CP was done compared with a delayed CP following surgical site infections [111]. Also there exists a correlation between treatment failure and the biomaterial used for reconstruction and various etiological factors like infection, flap breakdown, fixation protocol, and foreign body were identified along with the time frame of failure [100]. CP in the presence of a ventriculoperitoneal shunt (VPS) is associated with a higher rate of overall complications, including infection and bone resorption. Surgeons should consider staging these procedures when possible and counsel patients about these risks [112]. While formulating a treatment plan for reconstruction of cranial defects, one has to tailor make a strategy considering several factors such as systemic condition of the patient, status of the cranial surgical site, etiology behind craniectomy, choice of reconstruction material, duration from craniectomy and age of the patient. Despite the best efforts and ideal reconstruction attempts, failures remain a nagging reality (Figure 5). Table 7 summarizes the types of CP.

**Table 7:** Variation in patient outcomes in different types of cranioplasty VPS: Ventriculoperitoneal Shunt; TBI: Traumatic Brain Injury

Procedure name	Indication for surgery	Patient outcomes
Cranioplasty [97-100].	Reconstruction of cranial defects and restoration of structural integrity post- Decompressive craniectomy/craniotomy	Improved functional, cognitive, and neuropsychological outcomes; restoration of CSF fluid dynamics.
Immediate cranioplasty [102-105].	Neoplastic invasion and large mass effect or compression of the cranium	Reduced operating time and recovery; timely restoration of cranial structure integrity.
Delayed cranioplasty [102-105].	medically refractory intracranial hypertension (Increased ICP) or Intracranial infection after TBI	Potential reduction in complication rates; better management of infection post-TBI.
Cranioplasty with titanium mesh and polymethylmethacrylate Overlay [101,105].	Large cranial defects especially in adults	Can offer a durable and customizable reconstruction; potential increased risk of infection and complication rates.
Cranioplasty with autologous bone grafts [102].	Large cranial defects	Higher compatibility with the body; potential complications associated with harvesting of grafts and potential risk of bone resorption.
Cranioplasty in the presence of VPS [110].	Patients with Ventriculoperitoneal (VPS) Shunt	Higher rate of complications including post-operative infection and risk of bone resorption.

## Conclusions

Surgical Interventions, guided by classification modalities including both clinical and neuroimaging assessments, remain central in the management of TBIs and improvement of patient outcomes. This review focuses on the impact, effect of timing, risk factors, complications, and outcomes of various surgical interventions in contusions, hematoma evacuation, DC, ventriculostomy and cisternostomy for hydrocephalus, penetrating trauma surgical interventions, and cranioplasty. Although primary brain injury is often not fully reversible even by surgical interventions, prompt efforts at preventing secondary brain injury have been found to improve patient outcomes, reduce mortality, and optimize the cost of care.

The individual outcomes may vary depending upon a wide array of factors including the context of injury, timing of surgery, presence of foreign bodies, pre-operative and post-operative management, increased ICP and brain edema, CSF leaks, and patient-specific risk factors. Thus, each intervention requires a multidisciplinary approach, adhering to standardized guidelines and consideration of individual patient profiles including neurological and functional status, timing since injury, ICP, co-morbidities, and risk factors before devising an individualized plan for each patient. Future research should aim to optimize and refine these interventions, improve diagnostic protocols and strategies for rehabilitation and prompt management of possible complications, and enhance patient outcomes, recovery, and quality of life.
